# Association between level of suicide risk, characteristics of suicide attempts, and mental disorders among suicide attempters

**DOI:** 10.1186/s12889-018-5387-8

**Published:** 2018-04-11

**Authors:** Subin Park, Yeeun Lee, Tak Youn, Byung Soo Kim, Jong Ik Park, Haesoo Kim, Hyo Chu Lee, Jin Pyo Hong

**Affiliations:** 1 Department of Research Planning, Mental Health Research Institute, National Center for Mental Health, 127 Yongma-ro, Gwangjin-gu, Seoul, South Korea; 2 Department of Psychology, Korea University, 145 Anam-ro, Seongbuk-gu, Seoul, South Korea; 3 Department of Psychiatry, Dongguk University Ilsan Hospital, 27 Dongguk-ro, Ilsandong-gu, Goyang, South Korea; 4 Department of Psychiatry, Kyungpook National University, 807 Hoguk-ro, Buk-gu, Daegu, South Korea; 5 Department of Psychiatry, College of Medicine, Kangwon National University, 156 Baengnyeong-ro, Chuncheon, Gangwon-do South Korea; 6 Department of Psychiatry, Samsung Medical Center, 81 Irwon-ro, Gangnam-gu, Seoul, South Korea; 7 Department of Psychiatry, Samsung Medical Center, Sungkyunkwan University School of Medicine, 81 Irwon-ro, Gangnam-gu, Seoul, 06351 South Korea

**Keywords:** Suicide risk, Suicide attempt, Korea

## Abstract

**Background:**

Past attempted suicide is a strong predictor of future suicide risk, but the risk varies among suicide attempters. Hence, it is important to clarify distinguishing features of lifetime attempters with a high level of current suicide risk for efficient preventive management.

**Methods:**

We compared characteristics of suicide attempts and clinical characteristics among high-, moderate-, and low-risk attempters. Among the total of 6022 participants in the Korean Epidemiologic Catchment Area study, 193 reported a suicide attempt in their lifetime, 36 of which had high, 126 moderate, and 30 low levels of current suicide risk (1 incomplete response).

**Results:**

High-risk suicide attempters had more past attempts compared with moderate- and low-risk suicide attempters. Suicide attempts were closely linked to a wide range of psychiatric comorbidities regardless of degree of current level of suicide risk, but the relative risk for having at least one mental disorder was the highest in high-risk attempters. Specifically, the relative risks for depressive disorder, anxiety disorders including obsessive-compulsive disorder and post-traumatic stress disorder, and substance use disorders were higher in high-risk attempters, and relative risk for somatoform disorder was higher in low-risk attempters than others.

**Conclusions:**

Our findings indicated that special attention is required for suicide attempters with a history of repeated attempts and current mental disorders, particularly anxiety disorders.

## Background

History of suicide attempts is one of the strongest predictors of future suicide risk [1, 2]. Suicide attempts tend to recur with each attempt showing increased capability of lethality [3]. Almost half of serious suicide attempters make another attempt within 5 years [4], and the risk of suicide completion was about tens to hundreds of times higher than in the general population [5–7]. Thus, it is essential to monitor surviving suicide attempters to prevent future suicidal behaviors. However, future suicide risk varies among attempters given that not all past attempters repeat attempts and complete suicide [8]. Despite the relatively heightened risk, among individuals admitted to hospital for suicide attempts, 10–15% eventually complete suicide [9, 10], and, in a community-based sample, only 1.7% of participants with a history of attempts eventually completed suicide in the 17-year period [5]. Therefore, for efficient preventive management of past attempters, the multi-level factors associated with their increased suicide risks must be clarified.

Accumulated research has identified multiple factors that increase individuals’ suicide risk. Socio-demographically, higher suicide risk is linked to fewer years of education, marital status of single or divorced, unemployment, lower income, and family history of suicide [11–16]. The presence of any psychiatric disorder increases suicide risk, including depressive and bipolar, anxiety, impulse-control, substance, and personality disorders [1, 11, 16, 17], and having more disorders increases the occurrence of suicide attempt [13]. Key risk factors vary across the progression from suicide ideation to plan to attempt [12]. Unique associations between each psychiatric disorder and different stages of suicidal behaviors (ideation, plans, and attempts) were noted; depression was more linked to suicide ideation but not suicide plan or attempt among those experiencing suicide ideation (“suicide ideators”), and, in contrast, symptoms accompanied by anxiety and agitation or impulse-control difficulty were more linked to an increased risk of plan or attempt among ideators [13]. Fewer studies have examined the risk factors that predict future suicidal behavior or completed suicide in past attempters. Risk factors included unemployment, living alone, psychiatric morbidities, more number of attempts, suicide methods used (lethality), and suicide intent of previous attempt in suicide attempters [7–10, 18, 19].

However, this issue remains under-investigated using national studies from Asian countries; moreover, South Korea has a particularly high prevalence of suicide and suicidal behaviors compared to other countries [20] (i.e., the highest among Organization for Economic Cooperation and Development members [21]). Hence, we examined the association of suicide risk with a wide array of clinical, sociodemographic, and suicide-related variables among lifetime suicide attempters in a nationally representative sample of Koreans. We aimed to determine distinguishing features of high-risk suicide attempters as compared with moderate- and low-risk attempters.

## Methods

### Sample

We used data from the Korean Epidemiologic Catchment Area (KECA) study, which was conducted three times. In 2001 [22] and 2006 [23], the Korean version of the Composite International Diagnostic Interview (CIDI) was completed [24], and a follow-up to the KECA study was conducted in 2011. The KECA study was designed to evaluate the prevalence, correlates, and co-morbidities of mental disorders in the Diagnostic and Statistical Manual of Mental Disorders, Fourth Edition (DSM-IV) among Korean adults. Participants were selected using a stratified, multi-stage, clustered sample design based on a population census conducted by community registry offices in 2010 [25]. Individuals with the earliest birthday per selected household were randomly selected; 1682 of these refused to participate in the study, and, thus, 6022 individuals completed the face-to-face interview (78.7% response rate) [26]. The study protocol was approved by the institutional review board of the Seoul National University College of Medicine. Written informed consent was obtained from all participants before participation in the study.

### Measurement

#### Suicide risk

Suicide risk was assessed using the Suicide Module of the Korean version of the CIDI [24]. This module includes an assessment of the lifetime occurrence; age-of-onset; and latest occurrence of suicidal ideation, plans, and attempts [25, 27]. We initially selected lifetime suicide attempters with the following question: “Have you ever attempted suicide?” We then assessed the current suicide risk of the lifetime suicide attempters. Because the Suicide Module of the CIDI does not have a scoring system to assess level of suicide risk, we scored based on the scoring system of the Suicidality Module of the Mini International Neuropsychiatric Interview (MINI) using the corresponding items [28, 29]: a history of lifetime suicide attempt (4 points), suicide attempts with intent to die or expectation of death (9 points), suicide attempts without intent to die (4 points), suicidal ideation in the past month (6 points), suicide plans in the past month (8 points), and suicide attempts in the past month (10 points). We classified the level of current suicide risk based on the sum of points of the questionnaire we used (1–8 points: low, 9–16 points: moderate, ≥17 points: high). Among 193 participants who reported a suicide attempt in their lifetime, 36 were high-risk, 126 moderate-risk, and 30 low-risk. One participant was excluded from analysis due to an incomplete response.

#### Characteristics of suicide attempt

We collected information on the number of lifetime suicide attempts and family history of attempted and completed suicide. Precipitants for their attempted suicide were initially asked using the following dichotomous questions (i.e., Yes/No responses): Do you have any precipitant for attempted suicide? An individual with any precipitants for attempted suicide was asked to choose from the multiple choices of 1) aggravation of depression/anxiety, 2) conflict with family/friends/peers, 3) economic problems, 4) medical illness, 5) abuse/violence, 6) legal problems, 7) celebrity suicide, and 8) others [27].

#### Comorbidity of DSM-IV disorders

We used the CIDI [30] as a diagnostic assessment tool for psychiatric disorders. It is a fully structured diagnostic modality to establish psychiatric diagnoses based on definitions and criteria of the DSM-IV [31]. The Korean version of the CIDI [24] was later developed according to World Health Organization guidelines [32]. The inter-rater reliability, test/retest reliability, and validity of the Korean version of the CIDI showed kappa values ranging from 0.86 to 1.00, 0.42 to 0.89, and 0.50 to 1.00, respectively [25, 26]. We used psychiatric disorders in the past 12 months in this study.

#### Other variables

We used self-reported data from the 2011 KECA study for socio-demographic data, including gender, age, marital status, educational years, family income, marital status, and employment [26].

#### Statistical analysis

Weighted values were assigned to approximate the national population with respect to age and gender for each catchment area, using 2010 Korean census data, approved for use by the Korean National Statistical Office. We performed Pearson’s chi-square test to compare gender, marital status, economic status, family history of suicide, and precipitants among high-, moderate-, and low-risk suicide attempters. Independent t-test was used to compare age, years of education, age at first attempt, and number of attempts among high-, moderate-, and low-risk suicide attempters. Multivariate logistic regression analyses were performed to evaluate the odds ratios and 95% confidence intervals of 12-month comorbidities of mental disorders after controlling for age and gender. All statistical analyses were conducted using SPSS (version 21.0; SPSS Inc., Chicago, IL). A *p*-value < .05 was considered significant.

## Results

Table 1 summarizes the comparisons among high-, moderate-, and low-risk attempters. High-risk attempters had the highest numbers of suicide attempts, and low-risk attempters had the highest proportion of single attempts. There were no significant differences in family history and specific precipitants of suicide attempts by level of suicide risk.

**Table 1:** Comparison of Characteristics of Suicide Attempters by the Level of Suicidal Risk

Variables	High-risk attempters (*N* = 36)	Moderate-risk attempters (*N* = 126)	Low-risk attempters (*N* = 30)	Statistics		
				F	*p*	Post-hoc
Age	44.73 (15.31)	43.21 (15.77)	36.95 (13.73)	2.47	.087	
Education (years)	10.79 (3.96)	11.55 (4.10)	13.01 (3.00)	2.72	.068	
Age of first suicide attempt	32.92 (14.01)	27.45 (13.78)	26.16 (12.69)	2.59	.078	
No. of attempts	3.79 (5.25)	2.06 (1.90)	1.64 (1.04)	6.52	.002	High>Moderate>Low
	%	%	%	X^2^	*p*	Post-hoc
Gender, female	75.0	57.9	67.7	3.89	.143	
Divorced/separated/widowed	19.4	25.4	20.0	0.79	.675	
Family income, ≤1000$	62.7	57.9	52.9	0.87	.647	
Employment, employed	28.6	50.8	43.3	5.52	.063	
% of single attempt	36.1	56.0	63.3	5.85	.053	
% of family history of suicide attempt	20.0	15.4	13.3	0.60	.739	
% of family history of suicide	11.1	13.7	3.4	2.42	.298	
Precipitants						
Aggravation of depression/anxiety	36.1	25.4	25.8	1.66	.435	
Conflict with family/friends/peers	52.8	47.6	51.6	0.38	.826	
Economic problems	14.3	24.6	10.0	4.17	.124	
Medical illness	8.3	3.2	3.3	1.93	.381	
Other (i.e., abuse, violence, legal problem, celebrity suicide)	13.9	9.4	10.0	0.60	.741	

Table 2 indicates the adjusted odds ratios of mental disorders in high-, moderate-, and low-risk attempters after controlling for age and gender. A total of 83.3% of high-risk attempters had at least one mental disorder in the previous year, which fulfilled DSM-IV diagnostic criteria [24]. This prevalence was significantly higher than that of moderate- (46.0%) and low-risk attempters (36.7%). Anxiety disorder was the most common mental disorder in suicide attempters regardless of suicide risk degree. Overall, compared to non-attempters, post-traumatic stress disorder (PTSD) and somatoform disorder were significantly associated with all suicide attempters with different extents of current suicide risk. On the other hand, depressive disorder, obsessive-compulsive disorder (OCD), specific phobia, and nicotine and alcohol use disorder were associated with only high- and moderate-risk attempters but not with low-risk attempters. Further, relative risk was significantly higher for depressive disorder, anxiety disorder, and OCD in high-risk attempters than in moderate- and low-risk attempters. Relative risks for PTSD, specific phobia, and alcohol and nicotine use disorder were higher in high-risk attempters than in moderate-risk attempters. Relative risk for somatoform disorder was higher in low-risk attempters than in moderate-risk attempters.

**Table 2:** Associations Between Each Mental Disorder in the past 12 months and Level of Suicidal Risk

	High-risk attempters (*N* = 36)		Moderate-risk attempters (*N* = 126)		Low-risk attempters (*N* = 30)		RR (high/ low)	RR (high/ moderate)	RR (moderate/ low)
	%	AOR (95% CI)	%	AOR (95% CI)	%	AOR (95% CI)			
Depressive disorder	36.1	14.35 (7.43–27.72)^***^	15.6	5.88 (3.53–9.78)^***^	6.7	1.97 (0.45–8.71)	5.42 (1.33–22.14)	2.29 (1.27–4.15)	2.36 (0.58–9.56)
Anxiety disorder	50.0	17.13 (9.14–32.13)^***^	25.4	5.25 (3.44–8.02)^***^	16.7	2.54 (0.95–6.74)	3.00 (1.26–7.12)	1.97 (1.26–3.07)	1.52 (0.65–3.58)
PTSD	11.1	22.92 (8.25–63.64) ^***^	2.4	4.60 (1.33–15.93)^*^	3.3	9.06 (1.48–55.39)^*^	3.33 (0.39–28.25)	4.67 (1.09–19.90)	0.71 (0.08–6.63)
OCD	16.7	40.49 (15.46–106.06)^***^	4.8	12.24 (4.80–31.24)^***^	0.0		N/A	3.50 (1.20–10.20)	N/A
Specific phobia	30.6	9.47 (4.84–18.52)^***^	15.9	3.98 (2.41–6.59)^***^	10.0	1.94 (0.59–6.42)	3.06 (0.94–9.95)	1.93 (1.02–3.64)	1.59 (0.50–4.99)
Somatoform disorder	8.6	6.04 (1.94–18.81)^**^	3.2	2.80 (1.02–7.63)^*^	13.3	11.75 (3.88–35.60)^***^	0.63 (0.15–2.58)	2.63 (0.62–11.20)	0.24 (0.06–0.90)
Nicotine use disorder	23.6	13.14 (5.48–31.49)^***^	7.9	2.63 (1.33–5.23)^**^	6.7	3.13 (0.71–13.79)	3.43 (0.79–14.92)	2.88 (1.23–6.74)	1.19 (0.28–5.15)
Alcohol use disorder	27.8	15.31 (7.41–31.66)^***^	12.5	4.00 (2.28–7.02)^***^	10.0	2.89 (0.83–10.04)	2.78 (0.84–9.18)	2.20 (1.10–4.43)	1.26 (0.39–4.05)
Any mental disorder	83.3	28.77 (13.09–63.22)^***^	46.0	5.04 (3.53–7.22)^***^	36.7	3.42 (1.63–7.17)^**^	2.27 (1.39–3.72)	1.81 (1.43–2.30)	1.26 (0.76–2.08)

*AOR* odd ratio adjusted for sex and age, *PTSD* post-traumatic stress disorder, *GAD* generalized anxiety disorder, *OCD* obsessive compulsive disorder, *AOR* odd ratios adjusted for age and gender, *RR* Relative Risk

^***^, *p* < 0.001; ^**^, *p* < 0.01; ^*^. *p* < 0.05

## Discussion

In the present study, we investigated the association between level of suicide risk and characteristics of suicidal behaviors and mental disorders among lifetime suicide attempters. The results indicated that associated factors of suicide risk in attempters included number of lifetime suicide attempts and the presence of mental disorders.

The number of past suicide attempts varied across three groups of attempters in the order of high-risk > moderate-risk > low-risk attempters, suggesting that the suicidal problems of high-risk attempters are the most chronic. Through the habitual and repeated process of suicide attempt, high-risk attempters may have acquired the ability to engage in supposedly fear-provoking suicidal behaviors with increased tolerance for physical pain and decreased fear of death, which are conditions for lethal suicidal behaviors [3, 33].

Although all groups of attempters had a relatively heightened family history of attempted (13.3–20.0%) and completed suicide (3.4–13.7%), consistent with well-established association between individual’s suicidal behaviors and family history of suicide [34, 35], the family history did not differ by the extent of current suicide risk.

The close association of suicidal behaviors with a wide range of mental disorders is well-known [11, 17]. In line with prior findings, all lifetime attempters had a heightened risk of having one or more mental disorders. In particular, high-risk attempters had a relatively higher risk of any disorders with 83.3% presenting one or more diagnosable mental problems than other attempters. Among mental disorders, anxiety, depressive, and alcohol use disorders were the most prevalent among the suicide attempters in general, as reported in the US and cross-national findings [11, 12]. The highest prevalence of anxiety disorders among attempters is compatible with the findings that disorders featured by severe anxiety and agitation predicted the progression from suicide ideation to plan or attempt [13].

Comparing three groups of attempters having different suicide risk, high-risk attempters had particularly higher odds of depressive disorder and anxiety disorders as higher risk of suicide completion was noted for such disorders in Tidemalm et al. [8]. Among anxiety symptoms, higher current suicide risk was linked to higher risk of OCD and PTSD. A recent epidemiological study [36] found that individuals with OCD showed a greater risk of attempted suicide and suicide completion largely independent of other mental disorders. Similarly, PTSD was recently identified as one of the strongest predictors of suicidal behaviors [11, 37, 38]. Our findings further suggested that lifetime suicide attempters with OCD and/or PTSD tend to have a high current suicidal risk whereas the odds of somatoform disorder were relatively higher among low-risk attempters. The association between somatoform disorder and suicidal behaviors has gained relatively less attention, but studies have reported that somatoform disorder or medically unexplained physical pain were significantly related to suicidal ideation and suicidal behaviors [39, 40]. The current study added new findings that somatoform disorder is particularly more prevalent among lifetime suicidal attempters with low risk. Individuals with somatoform disorder may attempt suicide to get relief from distress associated with physical concerns or hopelessness derived from the belief that physical pain or concern would not improve [40], which could contribute to attempted suicide without strong desire to die. However, the underlying mechanism of high association of somatoform disorder with low-risk attempters was not addressed in the present study and requires further investigation.

The current study has several limitations. First, this was a cross-sectional study, and, thus, causal relationship between the degree of suicide risk and mental disorders could not be determined. Second, the degree of current suicide risk was not classified by standardized measurement. Additionally, since the study involved the administration of a largescale community survey, individual suicide behavior—ideation, plan, and attempt—was measured using a single-item and not standardized multi-item measures, which may limit the validity of measures. Lastly, suicide attempt may be under-reported because of stigma related to suicide attempts and concerns over its disclosure.

## Conclusions

Our findings indicated the differentially associated factors of Korean suicide attempters with high-, moderate-, and low-level risks. Special attention is required for individuals who present associated features, such as history of repeated suicidal attempts and overall anxiety disorders.

## Abbreviations

CIDI: Composite International Diagnostic Interview
DSM-IV: Diagnostic and Statistical Manual of Mental Disorders, Fourth Edition
KECA: Korean Epidemiologic Catchment Area
MINI: Mini International Neuropsychiatric Interview
NRF: National Research Foundation of Korea
OCD: Obsessive-compulsive disorder
PTSD: Post-traumatic stress disorder
